# Frequency and patterns of exposure to live poultry and the potential risk of avian influenza transmission to humans in urban Bangladesh

**DOI:** 10.1038/s41598-021-01327-x

**Published:** 2021-11-08

**Authors:** Isha Berry, Mahbubur Rahman, Meerjady Sabrina Flora, Amy L. Greer, Shaun K. Morris, Iqbal Ansary Khan, Sudipta Sarkar, Tanzila Naureen, David N. Fisman, Punam Mangtani

**Affiliations:** 1 Dalla Lana School of Public Health, University of Toronto, 155 College Street, Toronto, ON M5T 3M7 Canada; 2 Institute of Epidemiology, Disease Control and Research, Dhaka, Bangladesh; 3 Ontario Veterinary College, University of Guelph, Guelph, ON Canada; 4 Division of Infectious Disease and Center for Global Child Health, The Hospital for Sick Children, Toronto, ON Canada; 5 London School of Hygiene and Tropical Medicine, London, UK

**Keywords:** Risk factors, Epidemiology, Influenza virus

## Abstract

Avian influenza is endemic in Bangladesh, where greater than 90% of poultry are marketed through live poultry markets (LPMs). We conducted a population-based cross-sectional mobile telephone survey in urban Dhaka, Bangladesh to investigate the frequency and patterns of human exposure to live poultry in LPMs and at home. Among 1047 urban residents surveyed, 74.2% (95% CI 70.9–77.2) reported exposure to live poultry in the past year, with the majority of exposure occurring on a weekly basis. While visiting LPMs was less common amongst females (40.3%, 95% CI 35.0–45.8) than males (58.9%, 95% CI 54.0–63.5), females reported greater poultry exposure through food preparation, including defeathering (13.2%, 95% CI 9.5–17.9) and eviscerating (14.8%, 95% CI 11.2–19.4) (p < 0.001). A large proportion of the urban population is frequently exposed to live poultry in a setting where avian influenza viruses are endemic in LPMs. There is thus not only ample opportunity for spillover of avian influenza infections into humans in Dhaka, Bangladesh, but also greater potential for viral reassortment which could generate novel strains with pandemic potential.

## Introduction

Novel influenza strains with pandemic potential can emerge through zoonotic transmission from domestic or wild animals such as poultry and swine^1^. A majority of pandemic influenza strains in the past century have had an avian origin, making avian influenza viruses (AIVs) a particular global health concern^2–4^. Currently circulating AIV subtypes, which can be endemic in domestic poultry (e.g., H5N1, H7N9), have shown potential for reassortment with human influenza viruses under laboratory conditions^5–7^, and are a source of sporadic human infections^8^. The primary route of AIV transmission to humans is through direct or indirect exposure to live poultry, with high-risk practices including touching poultry, having poultry in the house, and preparing live poultry for consumption^9^. Live poultry markets (LPMs), which are known to play a critical role in maintaining and amplifying viruses, have also been identified as a source of AIV transmission between poultry species and from poultry to humans^9–11^.

Poultry production sectors in low- and middle-income countries, such as Bangladesh, are currently transitioning from small-scale backyard holdings to commercial production systems to meet growing consumer demands^12,13^. There is a growing body of evidence linking intensification practices to zoonotic disease emergence^14–16^. In Bangladesh, a variety of AIV subtypes (e.g., H5, H9, H10) have been isolated in LPMs^17,18^, and sporadic AIV poultry outbreaks have been reported since 2007^19^. In urban Bangladesh, where greater than 90% of poultry and poultry products are marketed through LPMs and mainly sold in an unprocessed form (i.e., live or freshly slaughtered)^19,20^, AIV exposure in the general population may be increasing^19,21^. Appropriate uptake of personal protective equipment (PPE) can reduce exposure thereby helping prevent zoonotic AIV transmission and reducing the risk of viral adaption to human hosts in the population^9^.

Although population-based poultry exposure surveys have been conducted in Hong Kong^22,23^, China^24–26^, Vietnam and Thailand^27^, such information is of interest in Bangladesh where there are lower resource capacities, different poultry production systems, and growing populations in dense urban areas. The extent of poultry exposure in the general urban population, as opposed to high-risk poultry sector workers in farms or markets^11,28,29^, has not been studied in Bangladesh. Furthermore, while in rural Bangladesh, females are generally involved in raising backyard poultry^30,31^, sex-disaggregated poultry exposures have not been explored in urban areas.

Here, we report on a population-based survey of live poultry exposure conducted among the general urban adult population of Dhaka, Bangladesh. We investigated the frequency and patterns of human exposure to live poultry in LPMs and at home, examined the uptake and adherence to prevention practices and use of PPE, and compared these patterns between males and females. This information may inform appropriate control strategies that are tailored to local socio-cultural contexts, and hence promote more sustainable mitigation measures to prevent transmission and emergence of influenza viruses at the human-poultry interface.

## Methods

### Study design and participants

We conducted a population-based cross-sectional survey in North and South Dhaka City Corporations (collectively known as DCC; population size: 6.97 million^32^), Bangladesh from September to November 2019. Details of the study procedures have been described elsewhere^33^. In brief, we employed a single-stage stratified mobile telephone survey using a computer-assisted telephone interview (CATI) system. We recruited an equal number of male and female participants to allow for robust sex-specific analyses. Individuals were eligible for inclusion if they were at least 18 years of age, current DCC residents, and had been residing in DCC for the previous year. Survey recruitment using the CATI system enabled random selection of mobile telephone numbers from sampling frames provided by each mobile telephone operator in Bangladesh. Telephone numbers were provided with the permission of the Bangladesh Telecommunication Regulatory Commission.

Each selected mobile telephone number was attempted up to four times, with calls made at different times of day and on different days of the week. At the time of first successful contact, respondents were screened for eligibility and sex before being invited to complete the survey. If the respondent was busy, the call was rescheduled for an alternate time within the next 7 days.

### Data collection

Data regarding live poultry exposures were collected using a questionnaire we developed, building on surveys conducted in urban China^24,25^; the survey instrument was translated into Bangla and pretested for length, content validity, and comprehension. The main settings for exposure to poultry in urban Bangladesh are LPMs, which are defined as a fixed collection of stalls or vendors where the general public can purchase live chickens, ducks, geese or any by-products of these in an unprocessed form^34^. In line with previous research, we define live poultry exposure as self-reported direct or indirect contact with live or unprocessed poultry at an LPM or in the home^24,25^. Information gathered included LPM visit rates in the past year, types of poultry purchased, contact (e.g., touching, proximity to defeathering and slaughtering) at LPMs, food preparation practices at home, prevention practices and PPE usage, recent influenza-like-illness (ILI), as well as individual and household socio-demographic characteristics. ILI was defined as reporting a new fever and cough in the past 10 days^35^. For participants not reporting visiting LPMs, we asked briefly about other household members’ live poultry exposures to estimate household-level exposure. Interviews were administered in Bangla over the phone by trained research assistants and data were recorded in real-time within the CATI system. The final survey instrument is available in the Supplementary Information.

### Statistical analysis

We applied post-stratification weights to adjust for differences in the distribution of age, sex, and education between the survey sample and the DCC demographic profile of the 2011 census^32^. Population demographics were evaluated using descriptive statistics, including proportions for categorical variables, stratified by sex. We estimated prevalence with 95% confidence intervals (CIs) for live poultry exposures and prevention practices stratified by sex. Differences in poultry exposure prevalence between males and females were examined using chi-square tests. Household-level poultry exposures and ILI were also estimated.

We applied a conservatively estimated number of visits to LPMs per response category using previously established methods^25,26^. Standardized midpoints per response category were assigned to obtain the overall and age/sex stratified mean annual number and standard deviation (SD) of LPM visits: 0 for no reported visits, 1 for 1–2 visits/year, 4 for 3–5 visits/year, 8 for 6–11 visits/year, 24 for 1–3 visits/month, 52 for 1–2 visits/week, 208 for 3–5 visits/week, 365 for daily visits.

All analyses were conducted in Stata 16.0 (StataCorp, College Station, TX, USA) using complex survey functions to incorporate survey weights.

### Ethics

All study methods were carried out in accordance with relevant guidelines and regulations, and this study received ethical approval from the committees of each of the participating research institutions: University of Toronto (Protocol No. 37657), the Institute of Epidemiology Disease Control and Research (IRB/2019/11) and the London School of Hygiene and Tropical Medicine (Ref. 17661). The study was conducted in accordance with the Declaration of Helsinki. All participants provided oral informed consent via telephone.

## Results

### Population characteristics

Between September and November 2019, we dialled 5486 unique mobile telephone numbers of which 2006 respondents were eligible^33^. Interviews were completed with 1047 participants, for an overall response rate of 52.2%. Of the 1047 participants, 16 (1.5%) were excluded from analysis due to missing information for survey weighting variables (i.e., age, sex, education). The demographic distribution of the weighted survey sample was generally representative of the DCC adult population^33^, with about 42% female, the majority aged under 35 years, and most with less than secondary education. Two thirds of females were home makers; about half of males had clerical, sales, and service jobs and another third were in skilled/unskilled labour occupations. Nearly half of participants lived in households of 4–5 members, with over two thirds living with children < 5 years of age. About 10% reported keeping live poultry in the household (Table 1).

**Table 1:** Demographic and household characteristics of participants, by sex, Dhaka City Corporation, Bangladesh.

	Male (%)	Female (%)	All (%)
Weighted sample^a^	n = 593	n = 438	n = 1031
**Individual characteristics**			
Age			
18–24	25.4	29.3	27.1
25–34	32.7	32.3	32.5
35–44	20.9	19.7	20.4
45–54	12.1	11.0	11.6
55–74	8.9	7.7	8.4
Education (highest completed)			
< Primary	20.8	28.1	23.9
Primary (year 5)	30.9	31.4	31.1
Secondary (year 10)	12.5	13.3	12.8
Higher secondary + (year 12)	35.8	27.2	32.2
Occupation^b^			
Professional/technical	4.9	2.0	3.7
Clerical, sales and service	48.9	9.9	32.2
Skilled/unskilled labour	32.4	16.1	25.4
Student	7.8	8.0	7.9
Home maker	0.5	63.5	27.3
Other^c^	5.5	0.6	3.4
Marital status^b^			
Single, never married	27.0	12.1	20.6
Married	71.2	78.7	74.4
Other^c^	1.8	9.2	4.9
Region^b^			
DCC North	50.3	60.5	54.6
DCC South	49.7	39.5	45.4
**Household characteristics**			
Household size^b^			
1–3 members	29.0	26.6	28.0
4–5 members	42.3	47.4	44.4
6 + members	28.7	26.0	27.6
Household with children < 5 years^b^			
Yes	73.2	64.5	69.5
No	26.8	35.5	30.5
Household keeps live poultry			
Yes	9.8	12.4	10.9
No	90.2	87.6	89.1

*CI* confidence interval, *yrs* years, *DCC* Dhaka City Corporation.

^a^Sample weighted by age, sex and education to the Dhaka City Corporation demographic profile of the 2011 Bangladesh census.

^b^Due to missing values, total weighted denominator for occupation n = 1024; marital status n = 1025; region n = 1009; household size n = 1025; children n = 1017.

^c^Other occupation includes retired and unemployed; other martial status includes widow/widower and divorced.

### Live poultry exposure and poultry purchasing practices

The overall prevalence of any live poultry exposure, including at LPMs or in the home during food preparation, in the past year was 74.2% (95% CI 70.9–77.2) (Table 2). At the household-level, 89.0% (95% CI 86.5–91.0) of participants reported that someone in their household and/or themselves visited an LPM. Additionally, of the 11.0% (95% CI 9.0–13.5) who did not report any household LPM visits, 32.3% (95% CI 22.6–43.8) purchased live poultry through mobile vendors.

**Table 2:** Exposure to live poultry in markets and during food preparation in the past year, by sex, Dhaka City Corporation, Bangladesh.

	Male	Female	All	p-value^a^
	% (95% CI)	% (95% CI)	% (95% CI)	
Weighted sample^b^	n = 593	n = 438	n = 1031	–
**Any poultry exposure (past year)**^c^				< 0.001
Yes	68.4 (63.7–72.7)	82.0 (77.6–85.7)	74.2 (70.9–77.2)	
No	31.6 (27.3–36.3)	18.0 (14.3–22.4)	25.8 (22.8–29.1)	
**Live poultry market-related exposure (past year)**				
Visited an LPM				< 0.001
Yes	58.9 (54.0–63.5)	40.3 (35.0–45.8)	51.0 (47.3–54.6)	
No	41.1 (36.5–46.0)	59.7 (54.2–65.0)	49.0 (45.4–52.7)	
Frequency of LPM visit^d^				0.830
$\ge$ 1–2/week	68.3 (62.3–73.8)	65.3 (56.5–73.2)	67.3 (62.4–71.9)	
1–3/month	25.1 (20.1–30.8)	27.2 (20.0–35.8)	25.8 (21.6–30.5)	
< 1/month	6.6 (4.1–10.4)	7.5 (4.2–13.2)	6.9 (4.8–9.9)	
Touched live poultry when buying^d^				< 0.001
Yes	55.2 (48.9–61.3)	36.5 (28.3–45.5)	48.9 (43.8–54.0)	
No	44.8 (38.7–51.1)	63.5 (54.5–71.7)	51.1 (46.0–56.2)	
Touched cages/basket when buying^d^				0.309
Yes	8.9 (6.0–13.0)	5.9 (2.9–11.6)	7.9 (5.6–11.0)	
No	91.1 (87.0–94.0)	94.1 (88.4–97.1)	92.1 (89.0–94.4)	
Slaughter location^d^				0.047
Market	89.7 (84.3–93.3)	96.7 (90.8–98.9)	92.0 (88.2–94.7)	
Home	10.0 (6.4–15.4)	2.9 (0.8–9.2)	7.6 (5.0–11.5)	
Other	0.3 (0.04–2.0)	0.5 (0.1–1.9)	0.3 (0.1–1.2)	
Stood near stall during slaughtering^e,f^				0.566
Yes	89.3 (84.5–92.7)	91.1 (85.6–94.6)	89.8 (86.4–92.6)	
No	10.7 (7.3–15.5)	8.9 (5.4–14.4)	10.1 (7.4–13.6)	
Stood near stall during defeathering^e,f^				0.008
Yes	85.6 (80.2–89.7)	94.6 (89.4–97.3)	88.5 (84.6–91.5)	
No	14.4 (10.3–19.8)	5.4 (2.7–10.6)	11.5 (8.5–15.4)	
Stood near stall during eviscerating^e,f^				0.570
Yes	77.3 (71.4–82.3)	80.1 (71.3–86.7)	78.2 (73.4–82.3)	
No	22.7 (17.7–28.6)	19.9 (13.3–28.7)	21.8 (17.7–26.6)	
**Food preparation related exposure (past year)**				
Slaughtered				< 0.001
Yes	25.7 (21.7–30.2)	8.2 (5.4–12.3)	18.3 (15.6–21.4)	
No	74.3 (69.8–78.3)	91.8 (87.7–94.6)	81.7 (78.6–84.4)	
Defeathered				< 0.001
Yes	5.8 (4.0–8.3)	13.2 (9.5–17.9)	8.9 (7.0–11.3)	
No	94.2 (91.7–96.0)	86.9 (82.1–90.5)	91.1 (88.7–93.0)	
Eviscerated				< 0.001
Yes	4.1 (2.5–6.5)	14.8 (11.2–19.4)	8.7 (6.8–11.0)	
No	95.9 (93.5–97.5)	85.2 (80.6–88.8)	91.3 (89.0–93.2)	
Cut/washed meat				< 0.001
Yes	19.3 (15.9–23.2)	77.9 (73.2–82.0)	44.2 (40.6–47.8)	
No	80.7 (76.8–84.1)	22.1 (18.0–26.8)	55.8 (52.2–59.4)	

*CI* confidence interval, *LPM* live poultry market.

^a^P-value obtained from chi-square test comparing males and females.

^b^Sample weighted by age, sex and education to the Dhaka City Corporation demographic profile of the 2011 Bangladesh census.

^c^Any poultry exposure is a combined outcome variable which includes both live poultry market-related and/or food preparation related exposures in the past year.

^d^Weighted denominator includes those who report visiting an LPM, n = 525.

^e^Weighted denominator includes those who report slaughter location as market, n = 454.

^f^Stood near defined as within 1 m, allowing for buyers to directly observe poultry processing.

**Figure 1:**
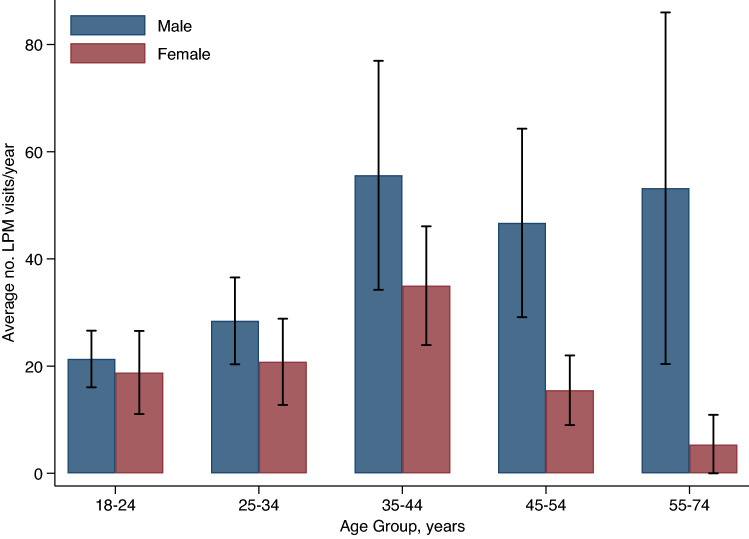
Live poultry markets visits reported in the past year, by sex and age group, Dhaka City Corporation, Bangladesh. Annual live poultry market visiting varied by age and sex. The average number of visits was consistently higher amongst males than females across all age groups, and the greatest number of visits were recorded amongst individuals aged 35–44 years in both sexes.

There were significant differences in poultry exposure between males and females (p < 0.001). Visiting LPMs was less common amongst females (40.3%, 95% CI 35.0–45.8) than males (58.9%, 95% CI 54.0–63.5). However, females had significantly greater poultry exposure through food preparation practices, with about three times as many females reporting defeathering (13.2%, 95% CI 9.5–17.9), eviscerating (14.8%, 95% CI 11.2–19.4), and cutting/washing fresh poultry meat (77.9%, 95% CI 73.2–82.0) as compared to males. Poultry slaughtering, when carried out at home as opposed to at the market, was more commonly reported by males (25.7%, 95% CI 21.7–30.2) than females (8.2%, 95% CI 5.4–12.3) (Table 2). The average number of LPM visits per year was estimated as 30.2 (SD 2.2); however, there was substantial variation by age and sex (Fig. 1). The average number of visits was consistently higher amongst males than females across all age groups, and the greatest number of visits were recorded amongst individuals aged 35–44 years in both sexes.

**Figure 2:**
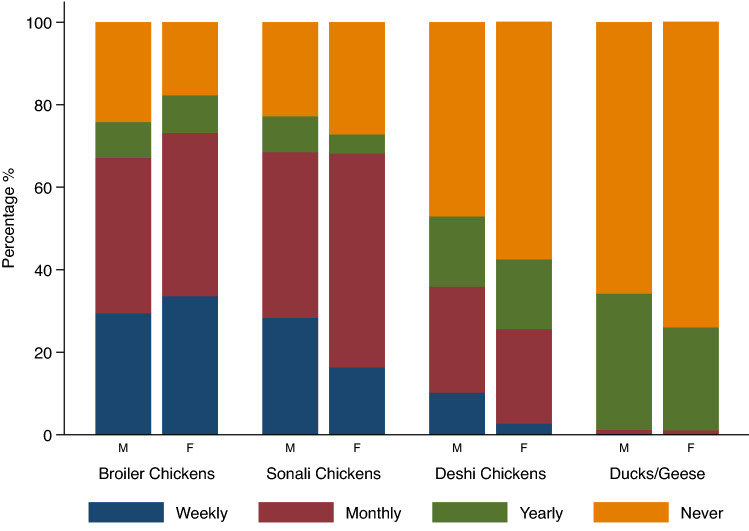
Poultry purchasing practices among those who have visited a live poultry market in the past year, by sex, Dhaka City Corporation, Bangladesh. Purchasing practices varied by poultry type, broiler and Sonali chickens were predominantly purchased on a weekly or monthly basis while ducks and geese were purchased only annually. Purchasing practices were similar between males and females. The denominator is the weighted number of respondents reporting visiting a market in the past year, by sex.

Of those who reported personally visiting an LPM in the past year, over two thirds (67.3%, 95% CI 62.4–71.9) made at least 1–2 visits per week with no observed difference between males and females (Table 2). About half of participants (48.9%, 95% CI 43.8–54.0) reported touching poultry while purchasing, with a significantly greater proportion of males than females reporting this (p < 0.001); 7.9% (95% CI 5.6–11.0) of participants reported indirectly contacting poultry (i.e., cages, baskets) when purchasing, with no notable difference by sex. Almost all respondents (92.0%, 95% CI 88.2–94.7) reported having their purchased poultry usually slaughtered at the market, and the majority reported standing by the stall during slaughtering, defeathering, and evisceration (Table 2). The most frequently purchased poultry types were broiler and Sonali chickens with the greatest proportion of respondents buying these on a weekly or monthly basis. Ducks and geese were purchased least frequently; types of purchases were similar between males and females (Fig. 2).

### Prevention practices

Among those who reported any poultry exposure, the majority (75.6%, 95% CI 71.6–79.2) reported always washing their hands with soap after exposure, with a greater proportion of females reporting this practice than males (Table 3). Use of PPE in the population was generally low, with most participants reporting never wearing facemasks (90.1%, 95% CI 87.4–92.2) when exposed to poultry. Amongst those who reported food preparation practices at home, the majority reported never wearing gloves (96.1%, 95% CI 94.1–97.4) or aprons (98.9%, 95% CI 97.8–99.4). These practices varied slightly by sex, with a significantly greater proportion of females than males reporting never wearing facemasks (p = 0.002). A further breakdown of prevention practices by source and location of exposure is detailed in Supplementary Table 1.

**Table 3:** Uptake of protective practices among those with poultry exposure in the past year, by sex, Dhaka City Corporation, Bangladesh.

	Male	Female	All	p-value^a^
	% (95% CI)	% (95% CI)	% (95% CI)	
Weighted sample^b^	n = 406	n = 359	n = 765	–
**Washed hands with soap**				< 0.001
Always	70.7 (65.0–75.8)	81.2 (75.3–85.9)	75.6 (71.6–79.2)	
Not always	21.4 (16.9–26.7)	18.1 (13.5–24.0)	19.8 (16.5–23.7)	
Never	8.0 (5.3–11.9)	0.6 (0.2–2.0)	4.5 (3.1–6.7)	
**Wore gloves**^c^				0.220
Always	0.9 (0.2–3.5)	3.0 (1.5–5.6)	2.1 (1.2–3.9)	
Not always	2.0 (0.8–4.9)	1.6 (0.8–3.3)	1.8 (1.0–3.1)	
Never	97.1 (93.9–98.7)	95.4 (92.6–97.2)	96.1 (94.1–97.4)	
**Wore facemask**				0.002
Always	3.9 (2.2–6.8)	1.0 (0.4–2.4)	2.5 (1.5–4.1)	
Not always	9.7 (6.9–13.5)	4.8 (2.9–8.0)	7.4 (5.6–9.8)	
Never	86.4 (82.1–89.8)	94.2 (91.0–96.3)	90.1 (87.4–92.2)	
**Wore apron**^c^				0.182
Always	0.4 (0.1–3.1)	0.3 (0.1–1.4)	0.4 (0.1–1.2)	
Not always	0.0	1.2 (0.5–2.8)	0.7 (0.3–1.7)	
Never	99.6 (96.9–99.9)	98.5 (96.8–99.3)	98.9 (97.8–99.4)	

*CI* confidence interval.

^a^P-value obtained from chi-square test comparing males and females.

^b^Sample weighted by age, sex and education to the Dhaka City Corporation demographic profile of the 2011 Bangladesh census. Weighted denominator is those who report any exposure to live poultry in the past year.

^c^Question was only asked to those who report slaughtering, defeathering, eviscerating and/or cutting poultry; weighted denominator includes only those who report these exposures, n = 566.

### Influenza like illness

Between September and November 2019, the cumulative incidence of reported ILI was 3.1% (95% CI 2.0–4.7). ILI varied by age, with those above age 45 years having the highest incidence, and those in age groups 25–34 and 35–44 years with the lowest (Supplementary Fig. 1). Amongst those with ILI symptoms, 32.7% (95% CI 15.5–56.1) reported exposure to live poultry in the 3 days before symptom onset, which might suggest that if epidemic influenza was circulating that co-infections with any AIVs may be possible. Household-level ILI cumulative incidence was 6.7% (95% CI 5.1–8.8).

## Discussion

This population-based cross-sectional survey provides empirical estimates of high levels of exposure to live poultry in DCC, Bangladesh, adding to our understanding of the potential risks of avian influenza transmission to humans in an urban low-income setting. Three quarters (74%) of the adult population are exposed to live poultry, with the majority reporting exposure on a weekly basis. Reported exposure practices included both LPM visits and at-home poultry food preparation but the patterns were different between males and females. While hygiene practices such as hand washing after poultry exposure was high, the use of PPE—which has also been recommended by the Government of Bangladesh for those who are in contact with live poultry^19^—was low, with less than 10% reporting wearing facemasks and less than 5% reporting wearing gloves or aprons. These results suggest that a large proportion of the urban population could easily be exposed to AIVs, which are known to circulate in LPMs in DCC, Bangladesh (H5 market prevalence: 21.6%, H9 market prevalence: 63.2%^10^)^17,18^.

In our study, 51% of the population reported personally visiting an LPM and 89% reported that at least one member of their household made a visit in the last year. While there are limited data on poultry exposure in other South Asian urban populations to enable comparisons, these estimates are considerably higher than those reported in other urban Asian settings such as in Hanoi, Vietnam where 34% of households reported buying live poultry^36^. Similarly, our results are higher than those of studies conducted in mainland urban China, which report between 19 and 45% of individuals visiting LPMs annually^24–26^. These differences could be due to greater perceived risks of AIVs in East and Southeast Asia, where there have been more recent and frequent AIV outbreaks^24,37^, as well as ecosocial and livelihood differences (e.g., less access to other fresh meat or cold chain supported processed meat outlets). Despite the high exposure to live poultry in urban Bangladesh, there have been a low number of reported human AIV cases (eight H5N1 cases to date^8^). While this could be due to underreporting of human AIV cases as well as moderate improvements in biosecurity practices at high-risk exposure points^19^, the rate of effective viral transmission of AIVs after close contact with infected poultry remains unclear and is perhaps lower than previously hypothesized.

Although contact with live poultry was high across the population, there were substantial variations in exposure practices between sex and age groups. Exposure amongst males was greatest through LPM visits while for females it was through food preparation—including defeathering and eviscerating, which while only reported by about 15% of females are both high-risk practices due to their considerable release of airborne AIV particles^38^. Our findings are similar to studies conducted in rural Bangladesh, which report that females have higher involvement in poultry evisceration, defeathering, as well as cutting and washing fresh meat^30,31^. Amongst those visiting LPMs, we found that almost half of respondents reported contact with poultry before slaughter including touching or picking up birds, which is similar to estimates in urban China^24,25^. The vast majority of LPM-goers also reported standing near stalls during slaughter, evisceration, and defeathering, which may result in exposure to airborne AIV particles^38^. While various biosecurity interventions such as designated slaughter areas have been implemented in markets to reduce direct contact with viscera^19^, our results suggest that there continues to be exposure risk within market settings in DCC, Bangladesh. Variations in exposure patterns are associated with differences in AIV risk^39^, and therefore public AIV awareness programs and interventions focused on structural changes in markets and behavioural risk modifications should be appropriately targeted to local sex-specific exposure patterns. These could include improved slaughter-house services at LPMs and greater uptake of bio-secure defeathering practices, such as buckets with lids, at homes for those reporting home-based poultry processing^40^.

Effective biosecurity measures can limit the risk of AIV transmission due to high-risk or frequent exposure^34^. We find that the overall uptake and adherence to prevention practices was low amongst those with poultry exposure. Hand hygiene practices were reported most, but adherence was still inadequate with 25% of respondents reporting never or not always washing their hands with soap after poultry exposure; however, this may have changed with the COVID-19 pandemic. Notably, reports of handwashing in our study are higher than those reported in previous studies that have used direct participant observation^41^. Potential interventions to increase handwashing practices could include the implementation of hand washing facilities within LPMs as has been effectively piloted in Indonesia^42^. Those who reported slaughtering, eviscerating, and/or defeathering poultry at home reported low PPE use, which is similar to uptake of prevention practices amongst LPM workers in Bangladesh^28^ and Nepal^43^. This is also in line with findings from a recent systematic review and meta-analysis, which found that overall prevalence of wearing gloves, facemasks, aprons, and boots was low within LPMs and even lower within households^9^. We found that amongst those self-reporting recent ILI symptoms almost a third reported poultry exposure in the three days prior to symptom onset, which would coincide with the influenza latent period^44^. Such instances of exposure between humans and poultry pose a risk for coinfection and genetic reassortment between influenza strains^45^, which could lead to the emergence of a novel influenza strain with pandemic potential. Public health programmes that ensure services and interventions are appropriately tailored to populations exposure, and improve the delivery, uptake, and adherence of PPE use and hygiene practices are required to reduce these risks.

Our study has some limitations. The survey response rate (52.2%) was slightly lower than previous telephone-based cross-sectional surveys conducted in Bangladesh using mobile phones^46,47^. We aimed to minimise this bias by using a robust sampling strategy, including multiple call attempts and call-rescheduling. We also weighted the sample by sex, age, and education to be broadly representative of the census urban population. Our population sampling frame also only included those with mobile phones, a source of selection bias if those with and without mobile phones have different poultry exposure practices. However, given that mobile phone ownership is greater than 87% in urban Bangladesh we anticipate this having minimal impact on population estimates^48^. Research in Afghanistan has found that those in lower socioeconomic quintiles had greater exposure to poultry^49^, which would in fact suggest our results could be underestimates. Greater understanding on if and how these patterns may have changed since the COVID-19 pandemic would be important and could be further examined in longitudinal or repeated cross-sectional surveys. Finally, this study is based on self-reported exposures and prevention practices, which could be influenced by social desirability bias. This may explain the reason for higher levels of handwashing than reported in previous studies conducted using direct observation^41^.

In conclusion, we find that exposure to live poultry is high and uptake of prevention practices are low among the general adult population in urban Bangladesh. There continue to be high risks for AIV transmission at human-poultry interfaces including at LPMs. Further research examining factors associated with poultry exposure and using this to develop interventions that are appropriately tailored to local socio-cultural contexts are needed to better support human and animal well-being. Strengthening collaborations between the human and animal health sectors is essential to better understand risk of transmission and protect populations from emergence of pandemic threats.

## Supplementary Information

Supplementary Information.

## Data Availability

The datasets analyzed during the current study are not publicly available due to pre-existing data sharing agreements. The data may be made available from the corresponding author on reasonable request.
