# Supplementary material for: Frequency and patterns of exposure to live poultry and the potential risk of avian influenza transmission to humans in urban Bangladesh
Source: Sci Rep. 2021 Nov 8;11:21880. doi: 10.1038/s41598-021-01327-x (PMC8575886; doi:10.1038/s41598-021-01327-x)
Supplement: Supplementary file 1 — Supplementary Information. [file 41598_2021_1327_MOESM1_ESM.docx]

**Supplementary Information**

**Title:** Frequency and patterns of exposure to live poultry and the potential risk of avian influenza transmission to humans in urban Bangladesh

**Authors:** Isha Berry^1^, Mahbubur Rahman^2^, Meerjady Sabrina Flora^2^, Amy L. Greer^1,3^, Shaun K. Morris^1,4^, Iqbal Ansary Khan^2^, Sudipta Sarkar^2^, Tanzila Naureen^2^, David N. Fisman^1^, Punam Mangtani^5^

**Author affiliations:**

^1^Dalla Lana School of Public Health, University of Toronto, Ontario, Canada

^2^Institute of Epidemiology, Disease Control and Research, Dhaka, Bangladesh

^3^Ontario Veterinary College, University of Guelph, Ontario, Canada

^4^Division of Infectious Disease and Center for Global Child Health, The Hospital for Sick Children, Ontario, Canada

^5^London School of Hygiene and Tropical Medicine, London, UK

**Supplemental Table 1.** Protective practices by source of exposure among those with poultry exposure in the past year, Dhaka City Corporation, Bangladesh

|  | **Male** | **Female** | **All** | **p-value^a^** |
| --- | --- | --- | --- | --- |
|  | **% (95% CI)** | **% (95% CI)** | **% (95% CI)** |  |
| **Weighted sample^b^** | **n= 406** | **n= 359** | **n= 765** | **-** |
| **Market** |  |  |  |  |
| Taking shoes off before entering home | 97.1 (94.2-98.6) | 98.7 (93.6-99.8) | 97.6 (95.5-98.8) | 0.342 |
| Washing hands with soap after entering home | 91.7 (87.5-94.6) | 76.7 (67.6-83.9) | 86.7 (82.5-90.0) | <0.001 |
| Wearing mask at the market | 15.0 (11.2-19.8) | 9.2 (5.4-15.2) | 13.1 (10.1-16.7) | 0.096 |
| **Slaughtering** |  |  |  |  |
| Wearing gloves | 0.4 (0.1-3.1) | 2.1 (0.3-14.0) | 0.8 (0.2-3.1) | 0.222 |
| Wearing mask | 2.4 (0.7-7.8) | 0.0 | 1.9 (0.6-6.4) | 0.431 |
| Wearing apron | 0.0 | 0.0 | 0.0 | - |
| Washing hands with soap | 90.6 (83.4-94.9) | 77.2 (43.8-93.7) | 88.4 (80.0-93.6) | 0.191 |
| **De-feathering** |  |  |  |  |
| Wearing gloves | 0.0 | 5.8 (1.7-18.4) | 3.7 (1.1-12.4) | 0.238 |
| Wearing mask | 2.3 (0.3-15.0) | 0.0 | 0.8 (0.1-6.0) | 0.189 |
| Wearing apron | 0.0 | 1.3 (0.2-9.0) | 0.9 (0.1-6.1) | 0.468 |
| Washing hands with soap | 100.0 | 100.0 | 100.0 | - |
| **Eviscerating** |  |  |  |  |
| Wearing gloves | 3.1 (0.4-19.4) | 5.2 (1.5-16.4) | 4.6 (1.5-13.0) | 0.659 |
| Wearing mask | 0.0 | 0.0 | 0.0 | - |
| Wearing apron | 0.0 | 1.2 (0.2-8.0) | 0.9 (0.1-6.1) | 0.551 |
| Washing hands with soap | 100.0 | 92.8 (79.3-97.8) | 94.9 (84.2-98.5) | 0.314 |
| **Cutting/Washing** |  |  |  |  |
| Wearing gloves | 4.9 (2.2-10.9) | 4.7 (2.9-7.5) | 4.7 (3.1-7.1) | 0.901 |
| Wearing mask | 2.3 (0.5-9.1) | 1.4 (0.6-3.2) | 1.6 (0.8-3.4) | 0.540 |
| Wearing apron | 0.8 (0.1-5.8) | 1.6 (0.7-3.2) | 1.4 (0.7-2.7) | 0.560 |
| Washing hands with soap | 96.8 (91.6-98.8) | 99.5 (98.2-99.9) | 98.8 (97.4-99.5) | 0.009 |
| **Note:** CI, confidence interval  ^a^P-value obtained from chi-square test comparing males and females. ^b^Sample weighted by age, sex and education to the Dhaka City Corporation demographic profile of the 2011 Bangladesh census. Participants reporting any exposure to live poultry in the past year, weighted denominators vary across variables based on those reporting each exposure behaviour. | | | | |

**Supplemental Figure 1.** Proportion of participants reporting influenza like illness, by age group, Dhaka City Corporation, Bangladesh. Denominator is the weighted number of respondents, by age group.

**Main Sections of Poultry Survey Questionniare:**

**Section 1. General Information**

*“I would first like to ask you some general questions about where you live in Dhaka City Corporation”*

| **Q. No** | **Question** | **Answer code** | **Instruction** |
| --- | --- | --- | --- |
| 1.1 | Try to assess the gender of the respondent  **If needed**: What is your gender? | Man……………………….............. **1**  Woman ……………………............**2**  Third Gender .........................**3**  Other................................….. **77**  Refuse......................……...….. **99** | Please avoid asking directly |
| 1.2 | What thana and area do you live in? | ………………………..Thana  Other................................….. **77**  Don’t Know.......................….. **88**  Refuse......................……...….. **99**  ………………………Area  Other................................….. **77**  Don’t Know.......................….. **88**  Refuse......................……...….. **99** | Record name of Thana and area in DCC (start typing and list of possible options will appear). If necessary ask relevant questions.  If other please record value in text box. |

**Section 2. Poultry Purchasing**

*“Now, I would like to discuss your usual poultry purchasing practices. By poultry we are referring to chickens, ducks, geese, etc.*

| **Q. No** | **Question** | **Answer code** | **Instruction** |
| --- | --- | --- | --- |
| 2.1a | Have you personally visited a live poultry market in the last year? | Yes ………...………….....….....**1**  No ……..............................**0**  Don’t Know..................... **88**  Refuse................……...….. **99** | **1**. Continue to 2.1b  **0/88/99**. Skip to 2.15 |
| 2.1b | Approximately how often did you visit live poultry markets in the last year? | Daily ............………...……....**1**  3-5 times/week ...............**2**  1-2/week ......................…**3**  1-3 times/month .............**4**  6-11 times/year ..............**5**  3-5/year ..........................**6**  1-2/year …...................…..**7**  Other ..............................**77**  Don’t Know..................... **88**  Refuse Answer...……...…...**99** | Choose option that is closest/best approximation  If other please record value in text box |
| 2.2 | What is the full name of the last (i.e. most recent) live poultry market you visited? | ……………………….................Market  Other ..............................**77**  Don’t Know.....................**88**  Refuse ................……...….**99** | Record market (start typing and list of possible options will appear).  If other please record value in text box. |
| 2.3a | Is that market known by any other names? | Yes ………...………….....….....**1**  No ……..............................**0**  Don’t Know..................... **88**  Refuse................……...….. **99** | **1**. Continue to 2.3b  **0/88/99**. Skip to 2.4 |
| 2.3b | What are the other names of that market? | ………………………..Market  Other ..............................**77**  Don’t Know..................... **88**  Refuse.................……...….. **99** | Record market (start typing and list of possible options will appear).  If other please record value in text box. |
| 2.4 | What thana is this market located in? | ………………………................Thana  Other ..............................**77**  Don’t Know.....................**88**  Refuse ................……...….**99** | Record name of Thana (start typing and list of possible options will appear).  If other please record value in text box. |
| 2.5 | About when did you visit this market? | ………………………................Days Ago  Don’t Know..................... **88**  Refuse.................……...….. **99** | Convert all options into days. I.e. 1 month ago = 30 days, 6 months ago = 180 days |
| 2.6a | Did anyone else from your household (including domestic helpers) go with you on this visit? | Yes ………...………….....….....**1**  No ……..............................**0**  Don’t Know..................... **88**  Refuse................……...….. **99** | **1.** Continue to 2.6b  **0/88/99.** Skip to 2.7a |
| 2.6b | About how old is that person? | 0-4 years………...………….......**1**  5-14 years………...………….....**2**  15-24 years………...………….**3**  25-34 years………...………….**4**  35-44 years………...………….**5**  45-54 years………...………….**6**  55-64 years………...………….**7**  65 or above………...………...**8**  Don’t Know..................... **88**  Refuse................……...….. **99** | If multiple individuals, record ages of each individual. |
| 2.7a | About how far is this market from your residence? | ……………………….............Kilometers  Don’t Know........................ **88**  Refuse ................…….....….. **99** | Record kilometers to 1 decimal place.  I.e. 1.0, 1.1, 1.2, etc. |
| 2.7b | Is this the market you usually visit in DCC? | Yes ………...………….....….....**1**  No ……..............................**0**  Don’t Know..................... **88**  Refuse................……...….. **99** | **1/88/99**. Skip to Q2.11  **0**. Continue to 2.7c |
| 2.7c | What is the full name of the market you usually visit? | …………………..............……..Market  Other ..............................**77**  Don’t Know.....................**88**  Refuse ................……...….**99** | Record market in DCC (start typing and list of possible options will appear).  If other please record value in text box. |
| 2.8a | Is the market you usually visit known by any other names? | Yes ………...………….....….....**1**  No ……..............................**0**  Don’t Know..................... **88**  Refuse................……...….. **99** | **1**. Continue to 2.8b  **0/88/99**. Skip to 2.9 |
| 2.8b | What are the other names of the market you usually visit? | ………………………..Market  Other ..............................**77**  Don’t Know.....................**88**  Refuse.................……...….**99** | Record market (start typing and list of possible options will appear).  If other please record value in text box. |
| 2.9 | What thana is the market you usually visit located in? | ………………………..Thana  Other ..............................**77**  Don’t Know.....................**88**  Refuse ................……...….**99** | Record name of Thana (start typing and list of possible options will appear).  If other please record value in text box. |
| 2.10 | About how far is the market you usually visit from your residence? | ………………………Kilometers  Don’t Know........................ **88**  Refuse ................…….....….. **99** | Record kilometers to 1 decimal place.  I.e. 1.0, 1.1, 1.2, etc. |
| 2.11a | Approximately how often have you personally purchased broiler chickens from live poultry markets in the last year? | Daily ............………...……....**1**  At least 1/week................**2**  About 1-2/month ..........…**3**  About 1-2/year ..............…**4**  Never................................**5**  Don’t Know..................... **88**  Refuse Answer...……...…...**99** | Choose option that is closest/best approximation |
| 2.11b | Approximately how often have you personally purchased sonali chickens from live poultry markets in the last year? | Daily ............………...……....**1**  At least 1/week................**2**  About 1-2/month ..........…**3**  About 1-2/year ..............…**4**  Never................................**5**  Don’t Know..................... **88**  Refuse Answer...……...…...**99** | Choose option that is closest/best approximation |
| 2.11c | Approximately how often have you personally purchased deshi chickens from live poultry markets in the last year? | Daily ............………...……....**1**  At least 1/week................**2**  About 1-2/month ..........…**3**  About 1-2/year ..............…**4**  Never................................**5**  Don’t Know..................... **88**  Refuse Answer...……...…...**99** | Choose option that is closest/best approximation |
| 2.11d | Approximately how often have you personally purchased ducks or geese from live poultry markets in the last year? | Daily ............………...……....**1**  At least 1/week................**2**  About 1-2/month ..........…**3**  About 1-2/year ..............…**4**  Never................................**5**  Don’t Know..................... **88**  Refuse Answer...……...…...**99** | Choose option that is closest/best approximation |
| 2.12a | Do you pick up/touch live poultry with your hands before you buy it? | Yes ………...………….....….....**1**  No ……..............................**0**  Don’t Know..................... **88**  Refuse................……...….. **99** | **1**. Continue to 2.12b  **0/88/99**. Skip to 2.13a |
| 2.12b | How often do you do that? | Always ............………...……....**1**  Usually.................................**2**  Rarely................................…**3**  Don’t Know........................ **88**  Refuse Answer...……...…......**99** |  |
| 2.13a | Do you touch cages/baskets containing live poultry with your hands before you buy it? | Yes ………...………….....….....**1**  No ……..............................**0**  Don’t Know..................... **88**  Refuse................……...….. **99** | **1**. Continue to 2.13b  **0/88/99**. Skip to 2.14a |
| 2.13b | How often do you do that? | Always ............………...……....**1**  Usually.................................**2**  Rarely................................…**3**  Don’t Know........................ **88**  Refuse Answer...……...…......**99** |  |
| 2.14a | Where do you usually slaughter live poultry after buying it? | Market ............………...……...**1**  Home...................................**2**  Other...................................**77**  Don’t Know........................ **88**  Refuse Answer...……...…......**99** | **1**. Continue to 2.14b  If other please record value in text box  **2/77/88/99**. Skip to Section 3 |
| 2.14b | Where in the market is the poultry usually slaughtered? | Stall ............………...…….........**1**  Slaughter Area.....................**2**  Other...................................**77**  Don’t Know........................ **88**  Refuse Answer...……...…......**99** | If other please record value in text box |
| 2.15a | **Only if 2.1a = 0** Has anyone else in your household (including domestic helpers) visited a live poultry market in the last year? | Yes ………...………….....….....**1**  No ……..............................**0**  Don’t Know..................... **88**  Refuse................……...….. **99** | **1**. Continue to 2.15b  **0/88/99.** Skip to 2.16 |
| 2.15b | **Only if 2.1a = 0 & 2.15a=1**  About how old is that person? | 0-4 years………...………….......**1**  5-14 years………...………….....**2**  15-24 years………...………….**3**  25-34 years………...………….**4**  35-44 years………...………….**5**  45-54 years………...………….**6**  55-64 years………...………….**7**  65 or above………...………...**8**  Don’t Know..................... **88**  Refuse................……...….. **99** | If multiple individuals, record ages of each individual. |
| 2.15c | **Only if 2.1a = 0 & 2.15a=1**  Approximately how often did that person visit live poultry markets in the last year? | Daily ............………...……....**1**  3-5 times/week ...............**2**  1-2/week ......................…**3**  1-3 times/month .............**4**  6-11 times/year ..............**5**  3-5/year ..........................**6**  1-2/year …...................…..**7**  Other ..............................**77**  Don’t Know..................... **88**  Refuse Answer...……...…...**99** | Choose option that is closest/best approximation  If other please record value in text box |
| 2.16a | **Only if 2.1a = 0 & 2.15a=0**  In the last year, where have you/your household acquired live poultry? | Mobile Vendor.....................**1**  Backyard poultry..................**2**  Don’t acquire.....................…**3**  Other...................................**77**  Don’t Know........................ **88**  Refuse Answer...……...…......**99** | **1.** Continue to 2.16b  **2/3/77/88/99**. Skip to Section 3  If other please record value in text box |
| 2.16b | **Only if 2.16 = 1**  Approximately how often did you buy live poultry from mobile vendors in the last year? | Daily ............………...……....**1**  3-5 times/week ...............**2**  1-2/week ......................…**3**  1-3 times/month .............**4**  6-11 times/year ..............**5**  3-5/year ..........................**6**  1-2/year …...................…..**7**  Other ..............................**77**  Don’t Know..................... **88**  Refuse Answer...……...…...**99** | Choose option that is closest/best approximation  If other please record value in text box |

**Section 3. Poultry Food Preparation**

*“Now, I would like to ask you a few questions about your poultry food preparation practices.”*

| **Q. No** | **Question** | **Answer code** | **Instruction** |
| --- | --- | --- | --- |
| 3.1a | Have you personally slaughtered or helped slaughter poultry in the last year? | Yes ………...………….....….....**1**  No ……..............................**0**  Don’t Know..................... **88**  Refuse................……...….. **99** | **1**. Continue to 3.1b  **0/88/99.** Skip to 3.1d if 2.14a=1 otherwise skip to 3.2a |
| 3.1b | Does anyone else from your household (including domestic helpers) help you with slaughtering? | Yes ………...………….....….....**1**  No ……..............................**0**  Don’t Know..................... **88**  Refuse................……...….. **99** | **1.** Continue to 3.1c  **0/88/99**. Skip to 3.1d if 2.14a=1 otherwise skip to 3.2a |
| 3.1c | About how old is that person? | 0-4 years………...………….......**1**  5-14 years………...………….....**2**  15-24 years………...………….**3**  25-34 years………...………….**4**  35-44 years………...………….**5**  45-54 years………...………….**6**  55-64 years………...………….**7**  65 or above………...………...**8**  Don’t Know..................... **88**  Refuse................……...….. **99** | If multiple individuals, record ages of each individual. |
| 3.1d | **Only if 2.14a=1**  When poultry is slaughtered at the market, do you stand near the stall and watch? | Yes ………...………….....….....**1**  No ……..............................**0**  Don’t Know..................... **88**  Refuse................……...….. **99** |  |
| 3.2a | Have you personally de-feathered or helped de-feather poultry in the last year? | Yes ………...………….....….....**1**  No ……..............................**0**  Don’t Know..................... **88**  Refuse................……...….. **99** | 1**.** Continue to 3.2b  **0/88/99**. Skip to 3.2e if 2.14a=1 otherwise skip to 3.3a |
| 3.2b | What tools do you usually use when de-feathering poultry? | Hand……..............................**1**  Machine.……........................**2**  Other................................. **77**  Don’t Know........................ **88**  Refuse ................…….....….. **99** | Select all that apply.  If other please record value in text box |
| 3.2c | Does anyone else from your household (including domestic helpers) help you with de-feathering? | Yes ………...………….....….....**1**  No ……..............................**0**  Don’t Know..................... **88**  Refuse................……...….. **99** | **1**. Continue to 3.2d  **0/88/99.** Skip to 3.2e if 2.14a=1 otherwise skip to 3.3a |
| 3.2d | About how old is that person? | 0-4 years………...………….......**1**  5-14 years………...………….....**2**  15-24 years………...………….**3**  25-34 years………...………….**4**  35-44 years………...………….**5**  45-54 years………...………….**6**  55-64 years………...………….**7**  65 or above………...………...**8**  Don’t Know..................... **88**  Refuse................……...….. **99** | If multiple individuals, record ages of each individual. |
| 3.2e | **Only if 2.14a=1**  When poultry is de-feathered at the market, do you stand near the stall and watch? | Yes ………...………….....….....**1**  No ……..............................**0**  Don’t Know..................... **88**  Refuse................……...….. **99** | **1.** Continue to 3.2f  **0/88/99.** Skip to 3.3a |
| 3.2f | **Only if 2.14a=1**  What tools are usually used for de-feathering at the stall? | By hand, with poultry not boiled/scalded before……...**1**  By hand, with poultry boiled/scalded before...............**2**  Uncovered machine, with poultry not boiled/scalded before ................**3**  Uncovered machine, with poultry boiled/scalded before......**4**  Covered machine, with poultry boiled/scalded before.....**5**  Other................................. **77**  Don’t Know........................ **88**  Refuse ................…….....….. **99** | If other please record value in text box |
| 3.3a | Have you personally eviscerated or helped eviscerate poultry in the last year? | Yes ………...………….....….....**1**  No ……..............................**0**  Don’t Know..................... **88**  Refuse................……...….. **99** | **1.** Continue to 3.3b  **0/88/99.** Skip to 3.3d if 2.14a=1 otherwise skip to 3.4 |
| 3.3b | Does anyone else from your household (including domestic helpers) help you with eviscerating? | Yes ………...………….....….....**1**  No ……..............................**0**  Don’t Know..................... **88**  Refuse................……...….. **99** | **1.** Continue to 3.3c  **0/88/99.** Skip to 3.3d if 2.14a=1 otherwise skip to 3.4 |
| 3.3c | About how old is that person? | 0-4 years………...………….......**1**  5-14 years………...………….....**2**  15-24 years………...………….**3**  25-34 years………...………….**4**  35-44 years………...………….**5**  45-54 years………...………….**6**  55-64 years………...………….**7**  65 or above………...………...**8**  Don’t Know..................... **88**  Refuse................……...….. **99** | If multiple individuals, record ages of each individual. |
| 3.3d | **Only if 2.14a=1**  When poultry is eviscerated at the market, do you stand near the stall and watch? | Yes ………...………….....….....**1**  No ……..............................**0**  Don’t Know..................... **88**  Refuse................……...….. **99** |  |
| 3.4a | Have you personally cut poultry into pieces or helped cut poultry into pieces in the last year? | Yes ………...………….....….....**1**  No ……..............................**0**  Don’t Know..................... **88**  Refuse................……...….. **99** | **1.** Continue to 3.4b  **0/88/99.** Skip to Section 4 |
| 3.4b | Does anyone else from your household (including domestic helpers) help you with cutting poultry? | Yes ………...………….....….....**1**  No ……..............................**0**  Don’t Know..................... **88**  Refuse................……...….. **99** | **1**. Continue to 3.4c  **0/88/99**. Skip to Section 4 |
| 3.4c | About how old is that person? | 0-4 years………...………….......**1**  5-14 years………...………….....**2**  15-24 years………...………….**3**  25-34 years………...………….**4**  35-44 years………...………….**5**  45-54 years………...………….**6**  55-64 years………...………….**7**  65 or above………...………...**8**  Don’t Know..................... **88**  Refuse................……...….. **99** | If multiple individuals, record ages of each individual. |

**Section 4. Protective Measures**

*“Now, I would like to ask you a few questions about your use of personal protective equipment during poultry handling.”*

| **Q. No** | **Question** | **Answer code** | **Instruction** |
| --- | --- | --- | --- |
| 4.1a | **Only if 2.1a =1**  After visiting a live poultry market do you take off your shoes before entering the house? | Yes ………...………….....….....**1**  No ……..............................**0**  Don’t Know..................... **88**  Refuse................……...….. **99** | **1**. Continue to 4.1b  **0/88/99**. Skip to 4.2 |
| 4.1b | How often do you do that? | Always ............………...……....**1**  Usually.................................**2**  Rarely................................…**3**  Don’t Know........................ **88**  Refuse Answer...……...…......**99** |  |
| 4.2a | **Only if 2.1a =1**  After visiting a live poultry market do you wash your hands immediately after returning home? | Yes ………...………….....….....**1**  No ……..............................**0**  Don’t Know..................... **88**  Refuse................……...….. **99** | **1**. Continue to 4.2b  **0/88/99**. Skip to 4.3 |
| 4.2b | How often do you do that? | Always ............………...……....**1**  Usually.................................**2**  Rarely................................…**3**  Don’t Know........................ **88**  Refuse Answer...……...…......**99** |  |
| 4.2c | Do you use soap when washing your hands? | Yes ………...………….....….....**1**  No ……..............................**0**  Don’t Know..................... **88**  Refuse................……...….. **99** | **1**. Continue to 4.2d  **0/88/99**. Skip to 4.3 |
| 4.2d | How often do you do that? | Always ............………...……....**1**  Usually.................................**2**  Rarely................................…**3**  Don’t Know........................ **88**  Refuse Answer...……...…......**99** |  |
| 4.3a | **Only if 2.1a =1**  When visiting live poultry markets do you wear a face mask? | Yes ………...………….....….....**1**  No ……..............................**0**  Don’t Know..................... **88**  Refuse................……...….. **99** | **1.** Continue to 4.3b  **0/88/99**. Skip to 4.4 |
| 4.3b | How often do you do that? | Always ............………...……....**1**  Usually.................................**2**  Rarely................................…**3**  Don’t Know........................ **88**  Refuse Answer...……...…......**99** |  |
| 4.4 | **Only if 3.1a=1**  During poultry slaughtering do you: |  |  |
| 4.4a | wear gloves? | Yes ………...………….....….....**1**  No ……..............................**0**  Don’t Know..................... **88**  Refuse................……...….. **99** | **1.** Continue to 4.4b  **0/88/99**. Skip to 4.4c |
| 4.4b | How often do you do that? | Always ............………...……....**1**  Usually.................................**2**  Rarely................................…**3**  Don’t Know........................ **88**  Refuse Answer...……...…......**99** |  |
| 4.4c | wear a face mask? | Yes ………...………….....….....**1**  No ……..............................**0**  Don’t Know..................... **88**  Refuse................……...….. **99** | **1**. Continue to 4.4d  **0/88/99.** Skip to 4.4e |
| 4.4d | How often do you do that? | Always ............………...……....**1**  Usually.................................**2**  Rarely................................…**3**  Don’t Know........................ **88**  Refuse Answer...……...…......**99** |  |
| 4.4e | wear an apron? | Yes ………...………….....….....**1**  No ……..............................**0**  Don’t Know..................... **88**  Refuse................……...….. **99** | **1**. Continue to 4.4f  **0/88/99.** Skip to 4.4g |
| 4.4f | How often do you do that? | Always ............………...……....**1**  Usually.................................**2**  Rarely................................…**3**  Don’t Know........................ **88**  Refuse Answer...……...…......**99** |  |
| 4.4g | wash your hands after? | Yes ………...………….....….....**1**  No ……..............................**0**  Don’t Know..................... **88**  Refuse................……...….. **99** | **1**. Continue to 4.4h  **0/88/99.** Skip to 4.5 |
| 4.4h | How often do you do that? | Always ............………...……....**1**  Usually.................................**2**  Rarely................................…**3**  Don’t Know........................ **88**  Refuse Answer...……...…......**99** |  |
| 4.4i | Use soap when washing your hands? | Yes ………...………….....….....**1**  No ……..............................**0**  Don’t Know..................... **88**  Refuse................……...….. **99** | **1**. Continue to 4.4j  **0/88/99.** Skip to 4.5 |
| 4.4j | How often do you do that? | Always ............………...……....**1**  Usually.................................**2**  Rarely................................…**3**  Don’t Know........................ **88**  Refuse Answer...……...…......**99** |  |
| 4.5 | **Only if 3.2a=1**  During poultry de-feathering do you: |  |  |
| 4.5a | wear gloves? | Yes ………...………….....….....**1**  No ……..............................**0**  Don’t Know..................... **88**  Refuse................……...….. **99** | **1**. Continue to 4.5b  **0/88/99**. Skip to 4.5c |
| 4.5b | How often do you do that? | Always ............………...……....**1**  Usually.................................**2**  Rarely................................…**3**  Don’t Know........................ **88**  Refuse Answer...……...…......**99** |  |
| 4.5c | wear a face mask? | Yes ………...………….....….....**1**  No ……..............................**0**  Don’t Know..................... **88**  Refuse................……...….. **99** | **1**. Continue to 4.5d  **0/88/99.** Skip to 4.5e |
| 4.5d | How often do you do that? | Always ............………...……....**1**  Usually.................................**2**  Rarely................................…**3**  Don’t Know........................ **88**  Refuse Answer...……...…......**99** |  |
| 4.5e | wear an apron? | Yes ………...………….....….....**1**  No ……..............................**0**  Don’t Know..................... **88**  Refuse................……...….. **99** | **1**. Continue to 4.5f  **0/88/99.** Skip to 4.5g |
| 4.5f | How often do you do that? | Always ............………...……....**1**  Usually.................................**2**  Rarely................................…**3**  Don’t Know........................ **88**  Refuse Answer...……...…......**99** |  |
| 4.5g | wash your hands after? | Yes ………...………….....….....**1**  No ……..............................**0**  Don’t Know..................... **88**  Refuse................……...….. **99** | **1**. Continue to 4.5h  **0/88/99.** Skip to 4.6 |
| 4.5h | How often do you do that? | Always ............………...……....**1**  Usually.................................**2**  Rarely................................…**3**  Don’t Know........................ **88**  Refuse Answer...……...…......**99** |  |
| 4.5i | Use soap when washing your hands? | Yes ………...………….....….....**1**  No ……..............................**0**  Don’t Know..................... **88**  Refuse................……...….. **99** | **1**. Continue to 4.5j  **0/88/99.** Skip to 4.6 |
| 4.5j | How often do you do that? | Always ............………...……....**1**  Usually.................................**2**  Rarely................................…**3**  Don’t Know........................ **88**  Refuse Answer...……...…......**99** |  |
| 4.6 | **Only if 3.3a=1**  During poultry eviscerating do you: |  |  |
| 4.6a | wear gloves? | Yes ………...………….....….....**1**  No ……..............................**0**  Don’t Know..................... **88**  Refuse................……...….. **99** | **1**. Continue to 4.6b  **0/88/99**. Skip to 4.6c |
| 4.6b | How often do you do that? | Always ............………...……....**1**  Usually.................................**2**  Rarely................................…**3**  Don’t Know........................ **88**  Refuse Answer...……...…......**99** |  |
| 4.6c | wear a face mask? | Yes ………...………….....….....**1**  No ……..............................**0**  Don’t Know..................... **88**  Refuse................……...….. **99** | **1**. Continue to 4.6d  **0/88/99.** Skip to 4.6e |
| 4.6d | How often do you do that? | Always ............………...……....**1**  Usually.................................**2**  Rarely................................…**3**  Don’t Know........................ **88**  Refuse Answer...……...…......**99** |  |
| 4.6e | wear an apron? | Yes ………...………….....….....**1**  No ……..............................**0**  Don’t Know..................... **88**  Refuse................……...….. **99** | **1**. Continue to 4.6f  **0/88/99.** Skip to 4.6g |
| 4.6f | How often do you do that? | Always ............………...……....**1**  Usually.................................**2**  Rarely................................…**3**  Don’t Know........................ **88**  Refuse Answer...……...…......**99** |  |
| 4.6g | wash your hands after? | Yes ………...………….....….....**1**  No ……..............................**0**  Don’t Know..................... **88**  Refuse................……...….. **99** | **1**. Continue to 4.6h  **0/88/99.** Skip to 4.7 |
| 4.6h | How often do you do that? | Always ............………...……....**1**  Usually.................................**2**  Rarely................................…**3**  Don’t Know........................ **88**  Refuse Answer...……...…......**99** |  |
| 4.6i | Use soap when washing your hands? | Yes ………...………….....….....**1**  No ……..............................**0**  Don’t Know..................... **88**  Refuse................……...….. **99** | **1**. Continue to 4.6j  **0/88/99.** Skip to 4.7 |
| 4.6j | How often do you do that? | Always ............………...……....**1**  Usually.................................**2**  Rarely................................…**3**  Don’t Know........................ **88**  Refuse Answer...……...…......**99** |  |
| 4.7 | **Only if 3.4a=1**  During cutting poultry into pieces do you: |  |  |
| 4.7a | wear gloves? | Yes ………...………….....….....**1**  No ……..............................**0**  Don’t Know..................... **88**  Refuse................……...….. **99** | **1**. Continue to 4.7b  **0/88/99**. Skip to 4.7c |
| 4.7b | How often do you do that? | Always ............………...……....**1**  Usually.................................**2**  Rarely................................…**3**  Don’t Know........................ **88**  Refuse Answer...……...…......**99** |  |
| 4.7c | wear a face mask? | Yes ………...………….....….....**1**  No ……..............................**0**  Don’t Know..................... **88**  Refuse................……...….. **99** | **1**. Continue to 4.7d  **0/88/99.** Skip to 4.7e |
| 4.7d | How often do you do that? | Always ............………...……....**1**  Usually.................................**2**  Rarely................................…**3**  Don’t Know........................ **88**  Refuse Answer...……...…......**99** |  |
| 4.7e | wear an apron? | Yes ………...………….....….....**1**  No ……..............................**0**  Don’t Know..................... **88**  Refuse................……...….. **99** | **1**. Continue to 4.7f  **0/88/99.** Skip to 4.7g |
| 4.7f | How often do you do that? | Always ............………...……....**1**  Usually.................................**2**  Rarely................................…**3**  Don’t Know........................ **88**  Refuse Answer...……...…......**99** |  |
| 4.7g | wash your hands after? | Yes ………...………….....….....**1**  No ……..............................**0**  Don’t Know..................... **88**  Refuse................……...….. **99** | **1**. Continue to 4.7h  **0/88/99.** Skip to Section 5 |
| 4.7h | How often do you do that? | Always ............………...……....**1**  Usually.................................**2**  Rarely................................…**3**  Don’t Know........................ **88**  Refuse Answer...……...…......**99** |  |
| 4.7i | Use soap when washing your hands? | Yes ………...………….....….....**1**  No ……..............................**0**  Don’t Know..................... **88**  Refuse................……...….. **99** | **1**. Continue to 4.7j  **0/88/99.** Skip to Section 5 |
| 4.7j | How often do you do that? | Always ............………...……....**1**  Usually.................................**2**  Rarely................................…**3**  Don’t Know........................ **88**  Refuse Answer...……...…......**99** |  |

**Section 5. Influenza**

*“Now, I would like to ask you a few questions about your health.”*

| **Q. No** | **Question** | **Answer code** | **Instruction** |
| --- | --- | --- | --- |
| 5.1a | In the past 10 days, have you had a fever? | Yes ………...………….....….....**1**  No ……..............................**0**  Don’t Know..................... **88**  Refuse................……...….. **99** | **1**. Continue to 5.1b  **0/88/99**. Skip to 5.4 |
| 5.1b | Was your fever measured at $\geq$100.4°F? | Yes ………...………….....….....**1**  No ……..............................**0**  Didn’t measure ................**2**  Don’t Know..................... **88**  Refuse................……...….. **99** |  |
| 5.1c | Was your fever accompanied by a cough? | Yes ………...………….....….....**1**  No ……..............................**0**  Don’t Know..................... **88**  Refuse................……...….. **99** |  |
| 5.1d | Was your fever accompanied by a sore throat? | Yes ………...………….....….....**1**  No ……..............................**0**  Don’t Know..................... **88**  Refuse................……...….. **99** |  |
| 5.2a | When did this illness start? | ………………………..Days Ago  Don’t Know..................... **88**  Refuse.................……...….. **99** | Convert all options into days. I.e. 1 week ago = 7 days |
| 5.3a | In the 3 days before you became unwell, did you visit a live poultry market or prepare poultry at home? | Yes ………...………….....….....**1**  No ……..............................**0**  Don’t Know..................... **88**  Refuse................……...….. **99** |  |
| 5.3b | Did you seek medical care for this illness? | Yes ………...………….....….....**1**  No ……..............................**0**  Don’t Know..................... **88**  Refuse................……...….. **99** | **1.**  Continue to 5.3c  **0/88/99**. Skip to 5.4 |
| 5.3c | Where did you seek medical care? | Hospital………...………….....….....**1**  Community Clinic....................**2**  Pharmacy................................**3**  MBBS Doctor..........................**4**  Village Doctor/Homeopath....**5**  Other....................................**77**  Don’t Know......................... **88**  Refuse................……...…...... **99** | If other please record value in text box |
| 5.4a | In the past 10 days, did anyone in your household (including domestic helpers) have a fever? | Yes ………...………….....….....**1**  No ……..............................**0**  Don’t Know..................... **88**  Refuse................……...….. **99** | **1**. Continue to 5.4b  **0/88/99**. Skip to Section 6  If multiple individuals, record for each individual |
| 5.4b | Was their fever measured at $\geq$100.4°F? | Yes ………...………….....….....**1**  No ……..............................**0**  Didn’t measure ................**2**  Don’t Know..................... **88**  Refuse................……...….. **99** |  |
| 5.4c | Was their fever accompanied by a cough? | Yes ………...………….....….....**1**  No ……..............................**0**  Don’t Know..................... **88**  Refuse................……...….. **99** |  |
| 5.4d | Was their fever accompanied by a sore throat? | Yes ………...………….....….....**1**  No ……..............................**0**  Don’t Know..................... **88**  Refuse................……...….. **99** |  |
| 5.5a | When did their illness start? | ………………………..Days Ago  Don’t Know..................... **88**  Refuse.................……...….. **99** | Convert all options into days. I.e. 1 week ago = 7 days |
| 5.5b | About how old is that person? | 0-4 years………...………….......**1**  5-14 years………...………….....**2**  15-24 years………...………….**3**  25-34 years………...………….**4**  35-44 years………...………….**5**  45-54 years………...………….**6**  55-64 years………...………….**7**  65 or above………...………...**8**  Don’t Know..................... **88**  Refuse................……...….. **99** | If multiple individuals, record ages of each individual. |
| 5.6a | In the 3 days before they became unwell, did they visit a live poultry market or prepare poultry at home? | Yes ………...………….....….....**1**  No ……..............................**0**  Don’t Know..................... **88**  Refuse................……...….. **99** |  |
| 5.6b | Did they seek medical care for this illness? | Yes ………...………….....….....**1**  No ……..............................**0**  Don’t Know..................... **88**  Refuse................……...….. **99** | **1.**  Continue to 5.6b  **0/88/99**. Skip to Section 6 |
| 5.6c | Where did they seek medical care? | Hospital………...………….....….....**1**  Community Clinic....................**2**  Pharmacy................................**3**  MBBS Doctor..........................**4**  Village Doctor/Homeopath....**5**  Other....................................**77**  Don’t Know......................... **88**  Refuse................……...…...... **99** | If other please record value in text box |

**Section 6. Demographics**

*“I have a few final questions to ask you about yourself and your household.”*

| **Q. No** | **Question** | **Answer code** | **Instruction** |
| --- | --- | --- | --- |
| 6.1 | What is your marital status? | Single ..............………...……....**1**  Married................................**2**  Divorced/Separated.............**3**  Widowed/Widower...........…**4**  Other.................................…**77**  Don’t Know........................ **88**  Refuse Answer...……...…......**99** | If other please record value in text box |
| 6.2 | What is the highest level of education you have completed (in years)? | ………………………..Years  No formal education...........**22**  SSC..................................... **44**  HSC.....................................**55**  Degree............................... **66**  Other.................................…**77**  Don’t Know........................ **88**  Refuse Answer...……...…......**99** | If no education/illiterate record 0.  Record only in completed years. I.e. If completed 7.5 years of education, write down 7. If necessary ask relevant questions. |
| 6.3 | What is your primary occupation (i.e. main source of income)? | Student...............……...…... **1**  Home Maker...............……..**2**  Government service...........**3**  Private service...............……**4**  Business Service..................**5**  Garment worker..................**6**  Teacher...............……...….... **7**  Doctor...............……...…........**8**  Engineer...............……...….... **9**  Lawyer...............……...…...... **10**  Army/Navy/Air...............……**11**  Tailor...............……...….........**12**  Public Representative..........**13**  Journalist...............……...…... **14**  Imam/priest/pope...............**15**  Domestic Helper...............…**16**  Agriculture...............……...…..**17**  Fisherman...............……...…...**18**  Poultry market worker...........**19**  Daily Labourer...............……..**20**  Other................……...…... **77**  Don’t know..................... **88**  Refuse................……...….. **99** | If other please record value in text box |
| 6.4 | Does your household keep live poultry (i.e. backyard poultry), or any other birds? | Yes ………...………….....….....**1**  No ……..............................**0**  Don’t Know..................... **88**  Refuse................……...….. **99** |  |
| 6.5a | How many people are living in your household (khana), including yourself? | ………………………..People  Don’t Know.......................….. **88**  Refuse......................……...….. **99** | Khana= Food from 1 pot  Record full number |
| 6.5b | Out of all people living your household, how many are children <18 years of age? | ………………………..People  Don’t Know.......................….. **88**  Refuse......................……...….. **99** | Record full number |
| 6.5c | Out of all people living in your household, how many are children <5 years of age? | ………………………..People  Don’t Know.......................….. **88**  Refuse......................……...….. **99** | Record full number |
